# Description of the largest cluster of tuberculosis notified in Norway 1997–2011: is the Norwegian tuberculosis control programme serving its purpose for high risk groups?

**DOI:** 10.1186/s12889-015-1701-x

**Published:** 2015-04-11

**Authors:** Bernardo R Guzman Herrador, Karin Rønning, Katrine Borgen, Turid Mannsåker, Ulf R Dahle

**Affiliations:** Division of Infectious Disease Control, Norwegian Institute of Public Health, Oslo, Norway; European Programme for Intervention Epidemiology Training (EPIET), European Centre for Disease Prevention and Control (ECDC), Stockholm, Sweden

**Keywords:** Tuberculosis, Control programme, Cluster, Elimination

## Abstract

**Background:**

Approximately 90% of new tuberculosis (TB) cases notified in Norway are asylum seekers and other immigrants from high-incidence countries. Asylum seekers are screened upon arrival at the National Immigration Centre. Other immigrants receive a letter from the Municipal Health Services requesting that they present for screening in their municipality of residence. In order to identify potential areas where the TB control programme could be better adapted for these groups, we studied the largest cluster of TB cases (“cluster X”) notified in Norway until 2011.

**Methods:**

Cases were defined as TB notifications reported to MSIS between January 1997 and December 2011 with identical IS*6110* RFLP assigned to cluster X. We described the cases in cluster X by using data from the Norwegian Surveillance System for Communicable Diseases (MSIS). Missing or incomplete information in MSIS was obtained from the National Reception Centre, Oslo University Hospital and Municipal Health services.

**Results:**

Of a total of 44 individuals meeting the case definition, 36 originated from Somalia and eight from other high-incidence countries. Twenty nine were asylum seekers and 15 were other immigrants. Upon arrival, 18/44 had been diagnosed with latent TB infection (LTBI), 9/44 tested negative for LTBI and 4/44 had been diagnosed with active TB. Results of TB-screening upon arrival were not available for the remaining 13/44 (one asylum seeker and 12 other immigrants). Five of the 12 other immigrants had still not been screened for TB after staying one year or longer in Norway.

**Conclusions:**

Most cases in cluster X with available results of TB-screening were already infected at arrival, indicating that their disease could be due to endogenous reactivation, rather than recent transmission after arrival to Norway. TB-status upon arrival was unknown for many of the other immigrants due to lack of initial screening. The reasons why conduction of the initial screening among other immigrants is failing should be explored and methods to simplify the TB screening at arrival should be implemented.

## Background

In 2012, the European Union/European Economic Area member states reported 68,423 tuberculosis (TB) cases (13.5 per 100,000 population) [1]. Norway has a low notification rate of TB (401 cases notified in 2012; around 8 cases per 100,000 inhabitants) [1,2]. The incidence of TB in Norway, as in many other industrialized countries, has steadily decreased in the native-born population during the last decades. Following increased immigration from high incidence countries, the number of reported cases in the foreign-born population has increased since the late 1980s [2,3], leading to an increase in the number of TB cases notified during the first decade of the 21^st^ century. TB transmission rates are however low, as the majority of TB cases are generated through re-activation of latent TB infection (LTBI) acquired abroad or domestically in a distant past [4]. In 2013, 86% of the TB cases in Norway were foreign-born, mainly from African countries [2]. Surveillance and screening of high risk groups is critical for the effective prevention and control of spread of TB in low-incidence countries and it is mandatory, including screening for LTBI, in Norway [5]. The WHO has developed a post-2015 Global TB Strategy, which was endorsed by the World Health Assembly in May 2014 [6]. The pillars and components of this strategy include management of LTBI in people with a high risk of developing active TB. This is considered one essential component if low-incidence countries are to eliminate TB. It has been shown that even in low-incidence countries certain groups are still at high risk of developing active TB. Therefore, TB control programmes should be adapted to identify and target these groups specifically [7].

In Norway, information on newly diagnosed TB cases is reported by medical specialists and microbiological laboratories to the Norwegian Institute of Public Health (NIPH), where the information is entered into the Norwegian Surveillance System for Communicable Diseases (MSIS). Clinical and laboratory data are linked in MSIS through the personal identification number of each case. All *Mycobacterium tuberculosis* isolates are sent to the National Reference Laboratory for Mycobacteria at the NIPH for confirmation and further characterization. Since 1994 all strains are genotyped using IS*6110* restriction fragment length polymorphism (RFLP), and since 2011 by use of mycobacterial interspersed repetitive units typing (MIRU). Cases are assigned a cluster number when identical strains are identified.

The Norwegian TB control programme was developed throughout the 20^th^ century and it has been revised regularly to be able to meet its purpose. As in many other low-incidence countries, the operability of the national TB control programme has been continuously challenged due to the increased immigration from high-incidence countries from numerous parts of the world followed by shift in the TB epidemiology, which has prompted revision of control programmes [8].

In this paper, we studied the largest cluster of TB cases (“cluster X”) notified in Norway until December 2011, with focus on screening procedures and results, in order to identify potential areas where the TB control programme could be better adapted to high-risk groups in low-incidence countries.

## Methods

### Routine TB surveillance and control among immigrants in Norway

Asylum seekers entering Norway are admitted to the National Reception Centre in Oslo for around three days where they undergo medical screening, including TB symptom screening, a tuberculin skin test and chest X-ray (for those 15 years or older). Suspected TB cases are referred to the Oslo University Hospital for diagnosis, clinical isolation and treatment. After initial screening, most asylum seekers move to local centres throughout the country. TB screening results are forwarded to municipal health services (MHS) in the municipality of residence for further follow up [9]. If the residence-application is rejected, the asylum seeker will not be deported while on treatment for active TB [4].

Other immigrants (refugees, students, workers, family reunion) from high-incidence countries [10] are also required by regulation to undergo TB screening. They receive a letter at their personal address from the MHS in which they are requested to contact the MHS for TB screening within four weeks [9]. The screening includes tests that are conducted on different days and at various locations.

The MHS are responsible for assessment and follow-up of TB screening results of asylum seekers and other immigrants, including both LTBI and active TB, and referring patients to the specialist health care for diagnosis and treatment. In order to facilitate such follow-up, all hospitals with TB care units have appointed one or more TB coordinators. In collaboration with medical expertise, they are responsible for developing personal treatment plans, individual follow-up and communication with all patients [9].

### Data gathering

Cases were defined as TB notifications reported to MSIS between January 1997 and December 2011 with identical IS*6110* RFLP assigned to cluster X. We selected the cases from the MSIS database and extracted the following variables: Age at time of diagnosis, sex, immigration reason (asylum seeker or other immigrant), country of birth, date of first arrival to Norway (month/year), date of first TB screening in Norway (month/year), results of TB screening: negative (Mantoux <6 mm; Pirquet ≤ 5 mm), positive (Mantoux ≥ 6 mm; Pirquet > 5 mm), or active disease, date of diagnosis (month/year), clinical presentation (pulmonary, lymph node, osteal, other).

Missing or incomplete information in MSIS was obtained from the historical records available at the National Reception Centre, Oslo University Hospital, or the individual TB coordinators.

We present the results section divided into “asylum seekers and “other immigrants” as the screening procedures are different in these two groups.

### Ethics statement

At NIPH, all *M. tuberculosis* strains are routinely collected for disease surveillance purposes. Also, the Norwegian Act relating to control of communicable diseases [11] obliges NIPH to monitor the TB situation within the country on a continuous basis, including collecting and collating case based epidemiological and microbiological information. For these reasons, ethical approval was not required for this study. All the epidemiological information analysed and included in the current manuscript is anonymized.

## Results

**Figure 1:**
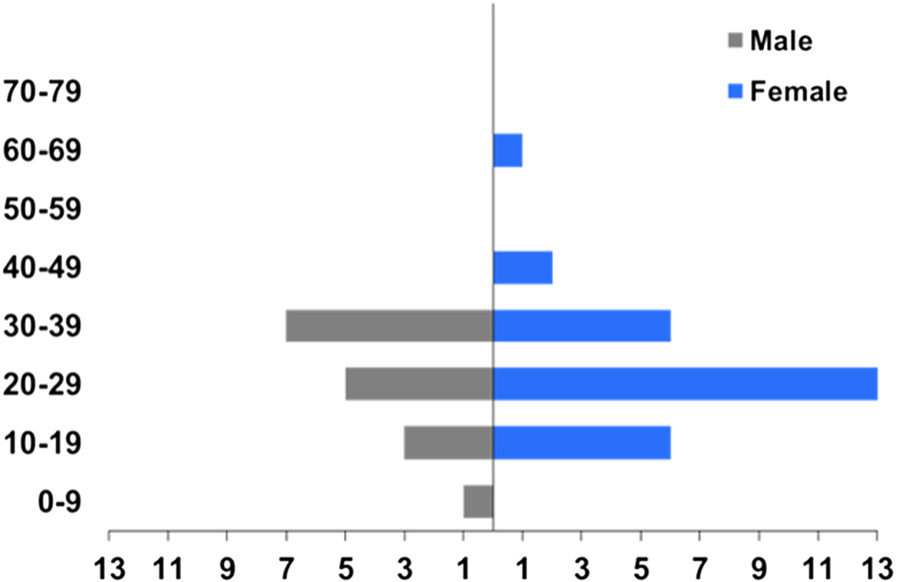
Distribution of TB cases by age and sex. Cluster X (n = 44); Norway, 1997–2011.

From January 1997 to December 2011, there were 4,367 TB notifications in MSIS, of which 3,449 were culture positive and 3,275 were analysed by IS*6110* RFLP. Of these 3,149 (96%) were unique or assigned to clusters with less than 10 individuals. A total of 44 cases were assigned to “cluster X”. Of these, 64% (28/44) were female and 93% (41/44) were below 40 years old (Figure 1). The number of cases notified each year ranged from one to six. Half of the cases (48%; 21/44) had pulmonary TB.

None of the cases were native-born Norwegians. The majority (82%; 36/44) was originally from Somalia followed by Ethiopia (two cases) and Ghana, Democratic Republic of Congo, Sudan, Tanzania, Thailand, and Romania, with one case each.

**Figure 2:**
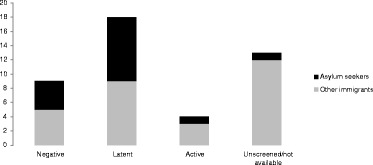
Flow chart with screening results at first arrival to Norway. Cluster X (n = 44); Norway, 1997–2011.

Upon arrival, 41% (18/44) had been diagnosed with latent TB infection (LTBI), 20% (9/44) tested negative for LTBI and 9% (4/44) were diagnosed with active TB. Results of TB-screening upon arrival were not available for the remaining 30% (13/44) (Figure 2). The median time from first arrival in Norway to diagnosis of active TB was 5.8 years for those who tested negative at arrival (range = 10 months-14 years; IQR = 10.3 years) and 5.4 years for those with unknown results (range = seven months-26.8 years; IQR = 12.3 years). Year of arrival was unknown for two cases.

### Asylum seekers

**Figure 3:**
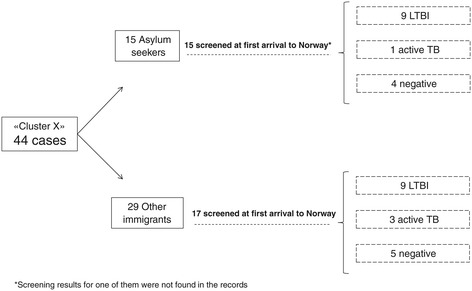
TB status at first arrival to Norway by type of immigrant. Cluster X (n = 44); Norway, 1997–2011.

One third (34%; 15/44) were asylum seekers who had been screened upon arrival to Norway at the National Reception Centre. Screening results were available for 93% of these (14/15). Most of them (64%; 9/14) had been diagnosed with LTBI, 29% (4/14) tested negative and 7% (1/14) had active TB (Figures 2 and 3). The median time from diagnosis of LTBI to the diagnosis of active TB for these nine cases was 2.6 years, ranging from two months to 8.4 years (IQR = 5.6 years).

### Other immigrants

Twenty nine cases (66% of all cases) were not asylum seekers and resided in 15 different counties at the time of TB diagnosis. Twelve of them (41%; 12/44) had not been screened during their first four weeks in Norway, and their TB status upon arrival was therefore unknown (Figures 2 and 3). Five of these had not been screened even after residing in Norway for one year or more. Among those with screening results available, 53%, (9/17) had LTBI at first arrival to Norway, 18% (3/17) were diagnosed with active TB, and 29% (5/17) tested negative. The median time from diagnosis of LTBI to the diagnosis of active TB was ten months, ranging from six months to 5.6 years (IQR = 1.2 years).

## Discussion

Most cases included in “cluster X” came originally from the same high-incidence region on the African continent and were already infected upon first arrival to Norway. However, although false-negative tuberculin skin tests results are well known and cannot be excluded [12], there are indications that cluster X also included individuals who may have become infected in Norway, as nine of them tested negative for LTBI at first arrival. Despite the possibility of TB transmission after arrival to Norway, such events are uncommon and largely limited to a few immigrant groups [13]. In addition, we have to bear in mind the possibility of relapse of a previously cured TB episode among those positive at first arrival after treatment completion. Previous studies conducted in Europe have concluded that, although the relapse rate is usually low, the risk is higher in neighborhoods with high TB incidence [14]. For this specific investigation we used surveillance data already collected in MSIS which did not allow determination of specific social relationships or geographical linkages within the cluster.

By looking in depth into the largest TB cluster reported in Norway we identified several specific areas for improvement in the TB control programme:

Most of the cases with known screening results had LTBI at first arrival and developed active TB several months or years later. In practice, a correct follow up of LTBI cases is not always achieved and some patients are not offered or do not accept treatment. In the current investigation we do not have data available on specific weaknesses in the follow up of single patient. Our results support the findings of a previous investigation that evaluated LTBI screening among asylum seekers in Norway, which concluded that a minor proportion of asylum seekers were treated as recommended for LTBI [15,16]. Reasons for this may be organizational factors affecting follow-up and referral, and specialists not following current guidelines [15,16].

Almost one third of the cases had not undergone the initial TB screening. The proportion of unscreened cases was considerably higher among immigrants other than asylum seekers. Many reasons, both logistical and cultural, may explain this, such as failure to be informed (i.e. failures in the administrative procedures, change of address, language-barriers), fear of possible deportation, lack of knowledge about public health consequences if not performed, or complex processes that involve different tests in different locations in new and unfamiliar communities.

Other low-incidence countries such as the United Kingdom and the United States have stressed the importance of screening and treatment both for LTBI and active TB in immigrants from high-incidence countries [17,18]. However, the on-going international debate regarding the cost-effectiveness of screening, targeting the correct groups and choice of screening methods [19,20] demonstrate that many low-incidence countries experience numerous challenges in adapting TB control programmes to the changing epidemiology. Still, compulsory TB screening for immigrants from high-incidence countries has been questioned, since most active disease develops after immigration and that early diagnosis has not been shown to convey public health benefits [21].

Our results cannot be considered a full evaluation of TB screening in Norway as cases in cluster X represent just a small subset of the total number of TB cases that occurred in Norway during the study period. However, we selected cluster X as our study population since it is by far the largest TB cluster identified in Norway and there is no reason to think that cases in this cluster have been screened differently upon entry than other TB cases in Norway.

## Conclusions

In conclusion, the description of this large TB cluster in Norway has clearly highlighted areas where the TB control programme could be better adapted for high risk groups. This experience may be valuable for other low-incidence countries and emphasizes that a large pool of latently infected people contributes to a growing proportion of future TB cases, unless effectively identified, treated or monitored. In Norway, a stronger collaboration between municipalities, together with a better flow of information between all stakeholders involved in the control programme could facilitate a closer individual follow-up of cases with LTBI at the first screening, preventing development of active TB. In addition, implementation of measures to simplify and facilitate access to TB screening at arrival (i.e. ensure that the letter is sent to the correct address in an understandable language to the relevant person; decrease as much as possible the number of different locations where different tests are conducted) could increase adherence of immigrants to the control programme.

TB elimination should remain an aim for low-incidence countries. This will however, require political commitment, financial support and high awareness of the challenges ahead. Full engagement will include the sharing of experiences, knowledge and expertise to develop strategies that are adapted to particular populations moving to and through these countries. If we are to eliminate TB in this country, the current example clearly highlights the need to provide additional outreach services to high risk groups. This experience is in accordance with the recommendations given by the 67th world health assembly the framework for tuberculosis elimination in low-incidence countries launched by the World Health Organization in 2014 [6,22].

## References

[1] European Centre for Disease Prevention and Control/WHO Regional Office for Europe (2014). Tuberculosis surveillance and monitoring in Europe 2014.

[2] Arnesen TM, Eide KÅ, Norheim G, Mengshoel AT, Sandbu S, Winje B (2014). Tuberkulose I Norge 2013. (Tuberculosis in Norway 2013. Yearly report).

[3] Winje B, Heldal E, Pettersen FO. Tuberculosis trends in Norway, 2002. Euro Surveill. 2003;7(42). http://www.eurosurveillance.org/ViewArticle.aspx?ArticleId=2309

[4] Dahle UR, Eldholm V, Winje BA, Mannsaker T, Heldal E (2007). Impact of immigration on the molecular epidemiology of Mycobacterium tuberculosis in a low-incidence country. Am J Respir Crit Care Med.

[5] forskrift om tuberkuloseontroll. Helse-og omsorgdepartementet; 2009. http://lovdata.no/dokument/SF/forskrift/2009-02-13-205.

[6] WHO Sixty-Seventh World Health Assembly (2014). Global strategy and targets for tuberculosis prevention, care and control after 2015. A67/11.

[7] Diel R, Loddenkemper R, Zellweger JP, Sotgiu G, D’Ambrosio L, Centis R (2013). Old ideas to innovate tuberculosis control: preventive treatment to achieve elimination. Eur Respir J.

[8] Story A, van Hest R, Hayward A (2006). Tuberculosis and social exclusion. BMJ.

[9] Forebygging og kontroll av tuberkulose: en veileder. Norwegian Institute of Public Health (In Norwegian); 2002. Available at http://www.fhi.no/dav/133D190B5E5C4E8393780C07DEE4CF7F.pdf.

[10] Countries with high occurrence of tuberculosis (Land med høy forekomst av tuberkulose). Norwegian Institute of public Health (In Norwegian). Available at http://www.fhi.no/eway/default.aspx?pid=239&trg=List_6212&Main_6157=6263:0:25,6568&MainContent_6263=6464:0:25,6580&List_6212=6218:0:25,8229:1:0:0:::0:0.

[11] Lov om vern mot smittsomme sykdommer [smittevernloven. (Norwegian Act relating to control of communicable diseases) available at http://lovdata.no/dokument/NL/lov/1994-08-05-55/KAPITTEL_7#§7-9.

[12] Centers for Disease Control and Prevention. Tuberculin skin testing. http://www.cdc.gov/tb/publications/factsheets/testing/skintesting.htm.

[13] Dahle UR, Sandven P, Heldal E, Caugant DA (2003). Continued low rates of transmission of Mycobacterium tuberculosis in Norway. J Clin Microbiol.

[14] Millet JP, Shaw E, Orcau A, Casals M, Miro JM, Cayla JA (2013). Barcelona Tuberculosis Recurrence Working G: Tuberculosis recurrence after completion treatment in a European city: reinfection or relapse?. PLoS One.

[15] Harstad I, Heldal E, Steinshamn SL, Garasen H, Winje BA, Jacobsen GW (2010). Screening and treatment of latent tuberculosis in a cohort of asylum seekers in Norway. Scand J Public Health.

[16] Harstad I, Jacobsen GW, Heldal E, Winje BA, Vahedi S, Helvik AS (2010). The role of entry screening in case finding of tuberculosis among asylum seekers in Norway. BMC Public Health..

[17] Linas BP, Wong AY, Freedberg KA, Horsburgh CR (2011). Priorities for screening and treatment of latent tuberculosis infection in the United States. Am J Respir Crit Care Med.

[18] Pareek M, Watson JP, Ormerod LP, Kon OM, Woltmann G, White PJ (2011). Screening of immigrants in the UK for imported latent tuberculosis: a multicentre cohort study and cost-effectiveness analysis. Lancet Infect Dis.

[19] Mandalakas AM, Menzies D (2011). Is screening immigrants for latent tuberculosis cost-effective?. Lancet Infect Dis.

[20] Pareek M, Baussano I, Abubakar I, Dye C, Lalvani A (2012). Evaluation of immigrant tuberculosis screening in industrialized countries. Emerg Infect Dis.

[21] Coker R (2004). Compulsory screening of immigrants for tuberculosis and HIV. BMJ.

[22] Framework for tuberculosis elimination in low-incidence countries. World Health organization; 2014. Available at http://apps.who.int/iris/bitstream/10665/132231/1/9789241507707_eng.pdf. 25473715

